# Preparation of Nanocellulose from Coffee Pulp and Its Potential as a Polymer Reinforcement

**DOI:** 10.1021/acsomega.3c02016

**Published:** 2023-07-06

**Authors:** Sataporn Malarat, Dilawin Khongpun, Kanokkorn Limtong, Napasin Sinthuwong, Pornpinun Soontornapaluk, Chularat Sakdaronnarong, Pattaraporn Posoknistakul

**Affiliations:** Department of Chemical Engineering, Faculty of Engineering, Mahidol University, Nakhon Pathom 73170, Thailand

## Abstract

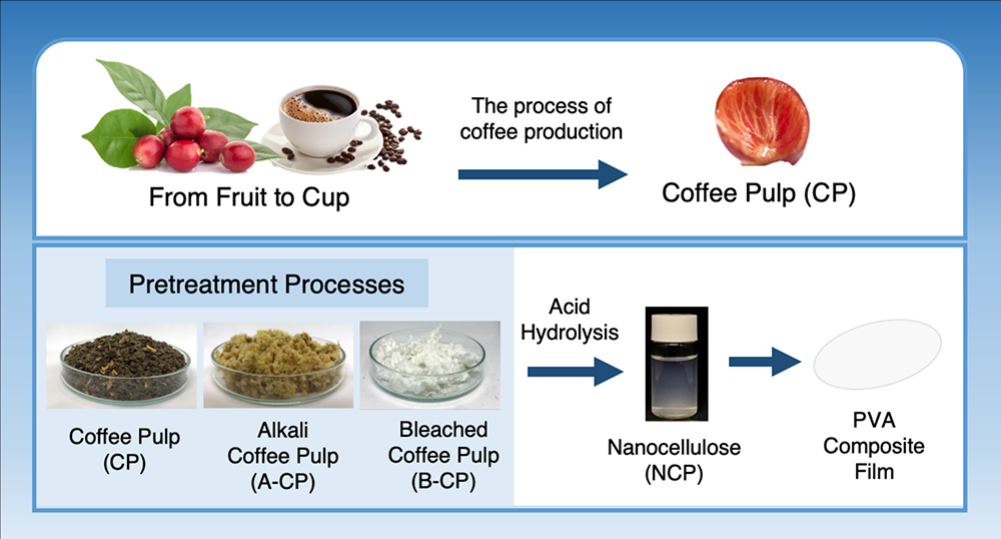

Coffee is one of the most valued agricultural products regarding its high commercialization rate. During the production of coffee beans, coffee pulp is obtained as one of the main byproducts with a cellulose content of more than 30% of dry weight. This research focused on the value-added potential of coffee pulp fiber as the reinforcement in composite materials. The nanocellulose coffee pulp (NCP) from the coffee pulp (CP) was prepared and subsequently used as a filler to reinforce the polyvinyl alcohol (PVA) matrix for the improvement of PVA composite properties. The CP was treated via alkali and bleaching treatment before the production of NCP using the acid hydrolysis treatment. The TEM result of NCP showed the successful preparation of NCP with an average diameter of 16.03 ± 4.70 nm with increasing crystallinity size and crystallinity index. The effect of glycerol (G) in the PVA matrix was observed. The result showed that glycerol had a play-role as a plasticizer for increased flexibility and decreased hardness and brittleness of PVA nanocomposite film. The nanocomposite film of PVA/G/NCP was fabricated with various ratios of NCP through the casting method. It was shown that the physical properties were improved with the presence of NCP in the PVA matrix compared to the neat PVA film.

## 1 Introduction

Due to the rising global demand for food, the recent extensive agricultural production may cause natural depletion and scarcity of resources. Regarding sustainable development goals (SDGs), the operation of agricultural production requires improvement to meet the sustainability standards to promote the benefit of overall sustainability. Coffee, as one of the world’s most prominent agricultural products, is a highly popular product consumed by millions of people every day. The high consumption of coffee has a huge impact on the environment due to the damage caused by the significant amount of waste generated during its production. The production of coffee generates many byproducts and residues during the processing from fruit to cup, such as coffee pulp (CP), coffee parchment, spent coffee grounds, and so forth. Coffee waste products and byproducts produced during coffee berry processing result in severe contamination and pose serious environmental problems in coffee-producing countries. On the other hand, the byproducts of coffee processing can be converted through chemical processes into valuable products, such as value-added chemicals or reinforcement agents in composite materials. Generally, cherry bean processing results in approximately 40–50% of the coffee pulp biomass. The production of coffee pulp has been estimated to be approximately 9.4 million tons/year.^1^ A previous study^2^ suggests that the main chemical composition of coffee pulp is approximately 55% of neutral detergent fiber, 31% of lignin, and 10% of ash. Among these compositions, the coffee pulp normally consumes 29% dry weight of the whole berry coffee.^3^

Cellulose is the world’s most abundant natural biopolymer with outstanding characteristics, including special morphology, crystallinity, high special surface area, nontoxicity, biodegradability, barrier properties, and high specific strength and modulus. Due to these characteristics, it has been used as the manufacturing material for several commodities in the food and medical industry. However, cellulose applications need to be expanded due to its hygroscopic nature and lack of melting properties.^4,5^ Several research studies have been done in isolating nanocellulose from cellulose to improve its properties and application in composite materials, and nanocellulose is a general term for cellulosic particles with nanoscale structural dimensions. In recent years, nanocellulose has gained increased interest for being used as a reinforcement material in biopolymer matrices, and the main features of NC are large specific surface area, high elastic modulus,^6^ high biocompatibility, and lower density, which are attractive properties.^7^ In addition, cellulose and nanocellulose are considered renewable materials extracted from a natural source, including agricultural residues. Incorporating lignocellulose, including nanocellulose, in biopolymers leads to improved biocomposite materials with better mechanical, barrier, and thermal properties, mainly required for packaging applications.^8−10^

Plastic is considered the most widely used polymer in our daily life. Most plastic is derived from fossil or petroleum feedstock and is difficult to degrade. Furthermore, nondegradable plastic or petroleum-based packaging generates huge post-consumer waste in the environment. It has also caused serious environmental impacts such as the depletion of natural resources, energy crises, global warming, and ecological problems.^11^ Increased and widespread environmental awareness, efforts of scientists, and engineering efforts are needed to replace petroleum-based materials that degrade the environment with biological materials. A biopolymer is used as an alternative for the partial replacement of conventional plastic due to its biodegradable and ecofriendly nature, solving the accumulation of persistent plastic waste to a certain extent.^12^ However, the biopolymer has a restricted potential for packaging materials, especially poorer thermal, mechanical, and barrier properties, compared to the nondegradable plastic material from petroleum.^13^ Incorporating nanofillers, including nanocellulose, into the biopolymer represents an effective way to improve the properties of the biopolymer and its other functions, including antimicrobial properties and oxygen-scavenging ability of the packaging material.^11^ Polyvinyl alcohol (PVA) is a biopolymer with unique nontoxic, semicrystalline, water-soluble, and hydrophilic properties. Moreover, it is also a very promising candidate for the preparation of biodegradable plastic. However, PVA has a hydrophilic nature and low resistance to humidity environment, reducing its oxygen barrier and mechanical properties. Therefore, packaging from the PVA biopolymer would be proper in the nonmoisture surrounding. To improve these properties, the PVA biopolymer is incorporated with other additive substances, including cellulose or nanocellulose.^4,14^

This research studies the possibility of converting cellulose from coffee pulp to nanocellulose. Furthermore, this study also investigated the effect of cellulose and nanocellulose from the coffee pulp as reinforcement materials on the PVA matrix using glycerol as a plasticizer. The effect of reinforcement on the thermal, mechanical, chemical, and optical properties was studied.

## 2 Experimental Section

### 2.1 Materials

The materials used in this study were prepared from coffee pulp (Bluekoff Co., Ltd., Thailand). The chemicals, including glycerol (Emsure) and polyvinyl alcohol (PVA) (Sigma), purchased were of commercial grade. Deionized water was received from the Faculty of Engineering, Mahidol University.

### 2.2 Preparation of Nanocellulose

#### 2.2.1 Coffee Pulp Pretreatment

Alkali treatment and bleaching treatment were done for the extraction of cellulose. Before the cellulose extraction, the ground coffee pulp (CP) was cleaned and dried in an oven at 60 °C for 48 h. The CP was treated with 4 w/v % NaOH solution under continuous stirring at 90 °C for 3 h. The ratio of CP:NaOH was 1:20 (w/v). The solid was washed and filtered with distilled water until the NaOH solution was removed before being dried in the oven at 60 °C for 16 h. In the bleaching treatment, the alkali-treated CP (A-CP) was bleached in the ratio of 1:20, with equal parts of acetate buffer solution, sodium chlorite (1.7 w/v %), and distilled water for 1 h under constant stirring at 80 °C. After that, the obtained solid was filtered and washed with distilled water several times until neutral pH was reached. It was then dried in the oven at 60 °C for 16 h.^15^

#### 2.2.2 Preparation of Nanocellulose from Coffee Pulp

**Figure 1:**
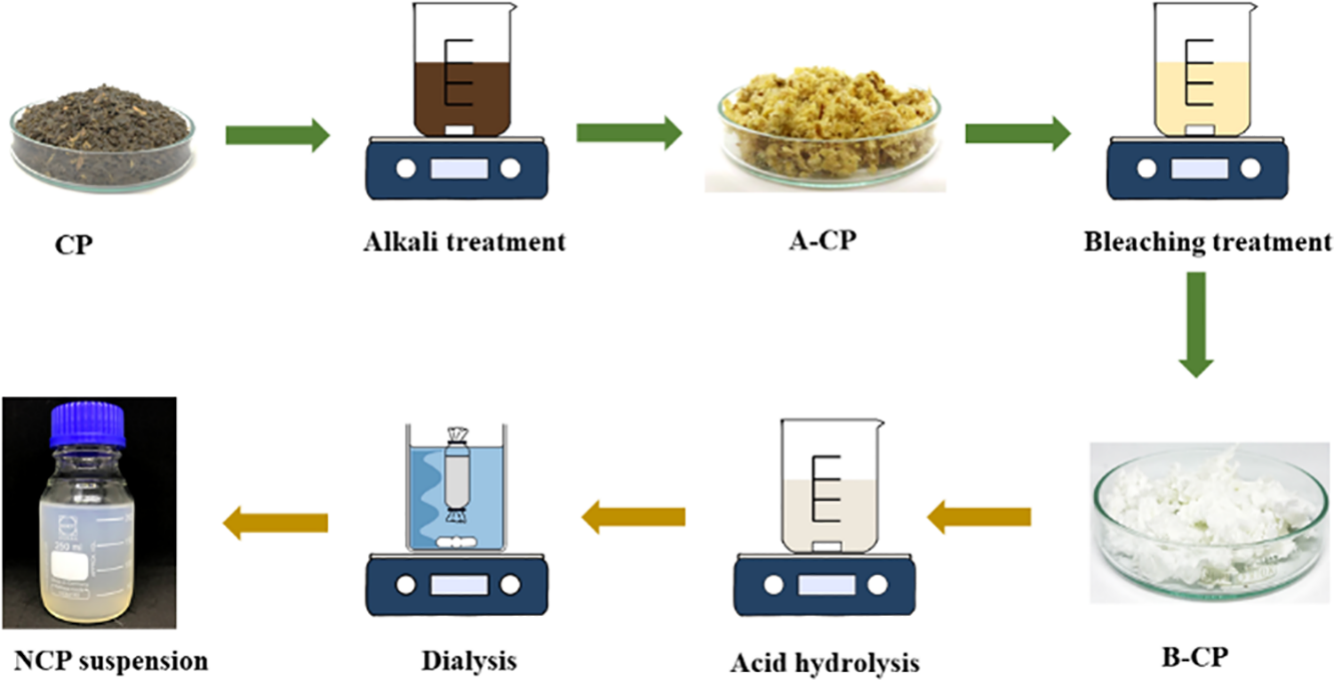
Schematic diagram of the preparation of NCP.

The nanocellulose coffee pulp (NCP) was prepared from bleach-treated coffee pulp (B-CP) via acid hydrolysis (Figure 1). The B-CP was hydrolyzed with 65 wt % of sulfuric acid in the ratio of bleached CP to sulfuric acid of 1:20; the reaction time was 1 h at room temperature under continuous stirring. The reaction was quenched by cold distilled water. First, the hydrolyzed B-CP was cooled. Then, it was centrifuged several times at 10,000 rpm for 10 min with deionized water. Afterward, the obtained turbid suspension was dialyzed with deionized water until constant pH was reached to obtain a neutral suspension free of sulfate ions. Before freeze-drying at −40 °C, the suspension was sonicated for 10 min to prevent the aggregation of particles.

### 2.3 Fabrication of Nanocellulose-Based PVA Composite Films

**Figure 2:**
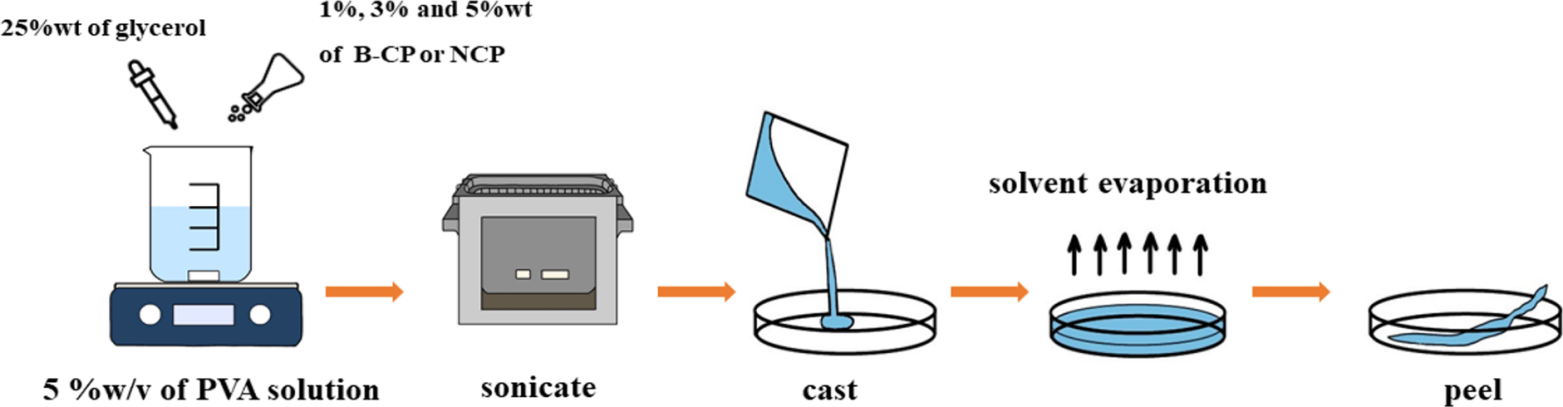
Schematic diagram of the preparation of PVA composite films.

Fabrication of composite films with B-CP, NCP, and glycerol was based on the solution casting method.^16^ A 5 w/v % of polyvinyl alcohol (PVA) solution was prepared by adding 0.5 g of PVA in 10 mL of DI water under constant stirring and heating up to 80 °C for 3 h (Figure 2). The obtained B-CP or NCP suspension (1, 3, and 5 wt %, relative to PVA mass) was transferred into PVA solution and stirred for 1 h. Next, the B-CP or NCP suspension was sonicated for 1 h before adding into the PVA solution. After adding the B-CP or NCP suspension, glycerol (25%, relative to PVA mass) as the plasticizer was also added. Then, the mixture was dispersed, homogenized, and its bubble removed via ultrasonication for 30 min. The final mixture was poured into a polystyrene Petri dish (90 mm × 15 mm) and dried at room temperature for 7 days after the dried film was dried again in the oven at 50 °C for 24 h.

### 2.4 Characterization

#### 2.4.1 Coffee Production Waste Chemical Composition Analysis

The chemical composition, including cellulose, lignin, hemicellulose, and ash, of coffee parchment and untreated and treated coffee pulp was determined^17^ by the calculation of neutral detergent fiber (NDF), acid detergent fiber (ADF), and acid detergent lignin (ADL). The concept of this approach is that plant cells can be divided into less digestible cell walls (hemicellulose, lignin, and cellulose) and highly digestible cell contents. NDF is a residue of insoluble neutral detergent solution (NDS), and ADF is an acid detergent solution (ADS) residue. The residue fraction after ADF treatment is not soluble in 72% sulfuric acid, which is ADL. Finally, the residue ADL fraction is calcined to be ash.^18^ The chemical composition is calculated by the following equation
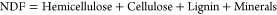

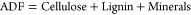

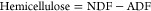
1
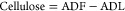
2
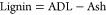
3

##### 2.4.1.1 Determination of Neutral Detergent Fiber

Seven grams of sodium borate decahydrate (Na_2_B_4_O_7_·10H_2_O), 19 g of disodium ethylene tetraacetate (EDTA), 30 g of sodium lauryl sulfate neutral (NaC_12_H_2_5SO_4_), 10 mL of 2-ethoxyethanol (C_4_H_10_O_2_), and 5 g of disodium phosphate (Na_2_HPO_4_) were mixed for the preparation of NDS. 1 g of ground coffee pulp was added into 100 mL of NDS with 0.5 g of sodium sulfite (Na_2_SO_3_) and heated to boiling for 1 h. After the onset of boiling, the sample was filtered using boiling water and acetone before drying at 105 °C for 8 h. The percentage of NDF was calculated by the remaining weight after NDF treatment.

##### 2.4.1.2 Determination of Acid Detergent Fiber

Acid detergent solution (ADS) is prepared from 20 g of cetyltrimethyl ammonium bromide and 50 g of sulfuric acid (H_2_S0_4_) in distilled water. 1 g of ground coffee pulp was added into 100 mL of ADS and heated to boiling for 1 h. After the onset of boiling, the sample was filtered using boiling water and acetone before drying at 105 °C for 8 h. The percentage of ADF was calculated by the remaining weight after ADF treatment.

##### 2.4.1.3 Determination of Acid Detergent Lignin and Ash Content

The remaining sample from ADF determination was added to 25 mL of 72% of sulfuric acid (H_2_SO_4_) with a continuous stir. The sample was filtered using boiling water and acetone before drying at 105 °C for 8 h. The percentage of ADL was calculated by the remaining weight after ADL treatment. Ash contents were determined by heating the residue from ADL for 2 h at 550 °C in a muffle furnace.

#### 2.4.2 Scanning Electron Microscopy

The microstructural analyses of the sample after different treatments, CP, A-CP, and B-CP, were performed using a scanning electron microscope (FEI/QUANTA 250), with an acceleration voltage of 15 kV. The surface samples were coated with gold in a vacuum sputter coater to provide electrical conductivity. All samples were ground into powder, dried in an oven at 60 °C for 24 h, and kept in a desiccator before characterization.

#### 2.4.3 Transmission Electron Microscopy

TEM was used to determine the morphology and size of NCP produced from CP. First, the NCP suspension was diluted and dropped on a carbon film supported by a carbon grid. TEM was performed using a JEOL system (JEM-2100 Plus, JAPAN), with an accelerating voltage of 200 kV. The diameter of NCP was measured in 100 samples, as recorded by ImageJ software.

#### 2.4.4 FTIR Spectroscopy

The FTIR spectra of all samples were used to examine the changes of the functional group that occurred by different treatments and the effect of B-CP or NCP filler on the PVA matrix with glycerol and without glycerol, based on the intensity and shift of the vibration band. The FTIR spectra of both fibers and films were recorded in the range of 4000–650 cm^–1^ by an FTIR spectrophotometer (Thermo Fisher Scientific, Nicolet 6700) under single-bounce mode (ATR). The PVA composite films were cut into small sizes, around 1 cm × 1 cm. Before characterization, all samples were dried in an oven at 60 °C for 24 h and kept in a desiccator.

#### 2.4.5 X-Ray Diffraction

XRD was used to study all specimens’ wide-angle X-ray diffraction patterns and crystallinity index (CI). It was performed using an X-ray diffractometer (PANalytical, Aris) with CuKα radiation (1.54 Å). The biomass sample was ground into powder, and the film sample was cut into 2 × 2 cm and placed on a holder. All of the samples were scanned in a 2θ range of 5–40° with a step size of 0.02°. The crystallinity index of CP, A-CP, B-CP, and NCP was calculated using the Segal method,^19^ following eq 4
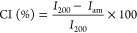
4where *I*_200_ is the maximum intensity of crystalline regions, and *I*_am_ is the minimum intensity of amorphous regions.

#### 2.4.6 Thermogravimetric Analysis

The TGA curve and DTG curve of all specimens were determined from a thermogravimetric analyzer (TGA 2, Mettler Toledo, Switzerland). About less than 10 mg of each sample was heated in the range of 25–850 °C at a heating rate of 10 °C/min under a nitrogen (N_2_) flow rate of 100 mL/min. The TGA curve provides the changes in the weight of a sample as a function of temperature, and DTG was the first derivative of the TGA curve. The percentage of weight loss in each sample was determined from the weight percentage of the residue during heating.

#### 2.4.7 Optical Properties

The light transmission of PVA composite films was measured using a UV–vis near-infrared (UV–vis–NIR) spectrometer, Perkin Elmer Lambda 95, with the UV–vis regions in the range of 200–800 nm. The result represented the relation of transmittance percentage (%*T*) and wavelength (nm).

#### 2.4.8 Mechanical Properties

Mechanical properties, including breaking stress (BS) or tensile strength (TS), elongation at break (EB), and elastic modulus (ED), were measured by an Instron testing machine (ASD8-82A.TSX), followed by ASTM D 882 E.mod at a load cell of 1 N. The dimensions of the film were as follows: length, 60 mm; width, 10 mm; and thickness, 0.04–0.06 mm range. Each film was measured over five films, exhibiting the result as an average value.

## 3 Results and Discussion

### 3.1 Chemical Composition of Coffee Pulp

The percentage of cellulose, hemicellulose, and lignin was determined with NDF, ADF, and ADL. The major components of coffee pulp and parchment are cellulose, lignin, and hemicellulose, as shown in Table 1. The chemical composition of coffee pulp comprises 31.26% cellulose, 17.32% lignin, and 7.25% hemicellulose. The chemical composition changes in coffee pulp through alkali treatment and bleaching treatment show that lignin, hemicellulose, and ash contents decreased as the amount of cellulose increased during the alkali treatment and bleaching treatment. Therefore, lignin and hemicellulose were attributed to the amorphous components.

**Table 1:** Chemical Composition of Coffee Pulp (CP), A-CP, and B-CP

material content	percent weight (wt %)		
	CP	A-CP	B-CP
cellulose	31.26	76.32	85.83
hemicellulose	7.25	5.87	3.71
lignin	17.32	10.98	1.56
ash	0.41	0.14	0.04

Moreover, the amorphous components’ reduction could also be observed from the color changes after pretreatment. After the alkali treatment, the color of the coffee pulp changed from brown to brownish yellow. Then, it turned white after the bleaching treatment due to the removal of lignin, hemicellulose, or noncellulose.^20^ The cellulose content of A-CP and B-CP increased to 76.32% and 85.83%, respectively. Correspondingly, the lignin content declined from 17.32 to 1.56%, the hemicellulose content declined from 7.25 to 3.71%, and the ash content declined from 0.4 to 0.04%. These results represent that the chemical treatment with NaOH and NaClO_2_ efficiently eliminated noncellulose from the coffee pulp to obtain pure cellulose. The change in the chemical composition of CP during all treatments results in the difference in morphologies, chemical structures, crystallinity, and thermal properties of the CP fiber.

### 3.2 Preparation of Nanocellulose from Coffee Pulp

#### 3.2.1 Morphology of the Prepared Nanocellulose

**Figure 3:**
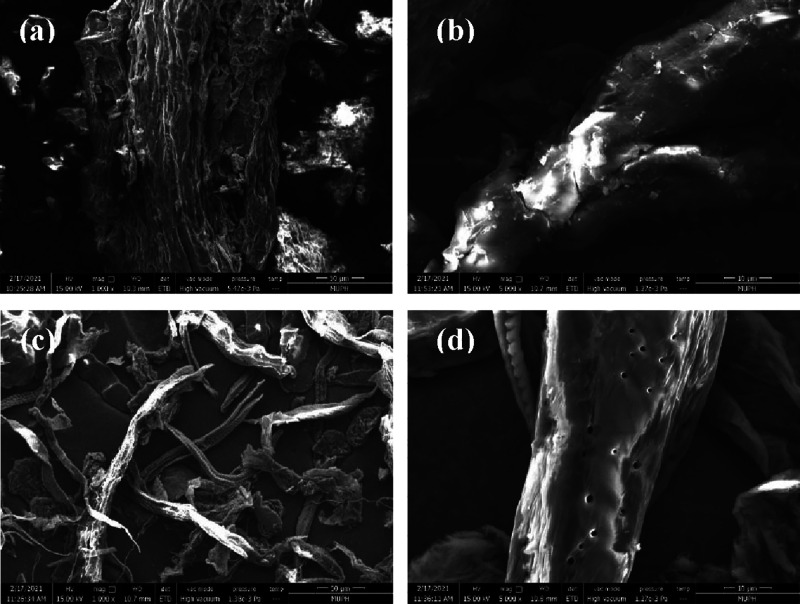
SEM micrographs (a) CP, (b) A-CP, (C) B-CP 1000×, and (d) B-CP 5000×.

The morphology of CP surfaces via different chemical treatments was investigated by using SEM, as shown in Figure 3. It was reported that after the chemical treatment, the obtained oil palm mesocarp fiber revealed its surface due to the elimination of noncellulose, macromolecular weights such as that of pectin and wax lignin, hemicellulose, and the other impurities from the untreated fiber.^21^ As shown in Figure 3, significant changes in the CP morphology can be noticed after alkali and bleaching treatments. The surface morphology of A-CP became cleaner and smoother due to impurities. In alkali treatment, substances such as pectin and wax were removed from the A-CP surface due to the interaction with sodium hydroxide (NaOH).^22^ The bleaching treatment starts to separate bundle fibers into individual fibers with various diameters. The surface of the B-CP obtained after bleaching with NaClO_2_/acetate buffer was found to be cleaner and smoother than that of A-CP. In addition, the B-CP surface appeared microporous linear on microfibrils.

**Figure 4:**
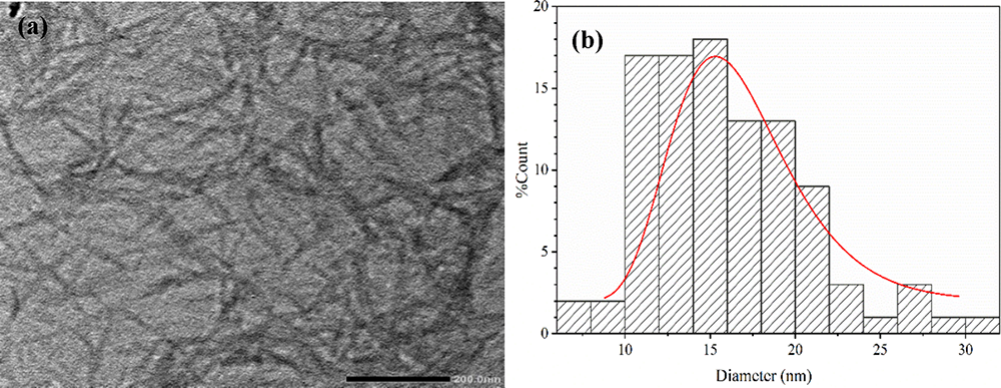
TEM micrographs of (a) NCP and (b) diameter histograms of NCP.

TEM images were used to investigate the nanomaterial morphology and nanometer scale. Figure 4 shows the morphology and size of the fiber after acid hydrolysis with sulfuric acid. This micrograph showed that the obtained nanocellulose (NCP) in the form of cellulose nanocrystals has a rodlike or needle-like shape with a diameter of 16.03 ± 4.70 nm, confirming its successful extraction from the coffee pulp.^23^ Fibers upon hydrolysis appeared finer and tended to agglomerate and stack crystallites together due to the penetration of hydronium ions from H_2_SO_4_ in an amorphous structure. As a result, the glycosidic bond in the amorphous structure was cleaved during hydrolysis. Therefore, microfibers were divided from bunched fibers and decreased into smaller sizes.^24^

#### 3.2.2 Chemical Structure of Nanocellulose

##### 3.2.2.1 FTIR Spectroscopy

**Figure 5:**
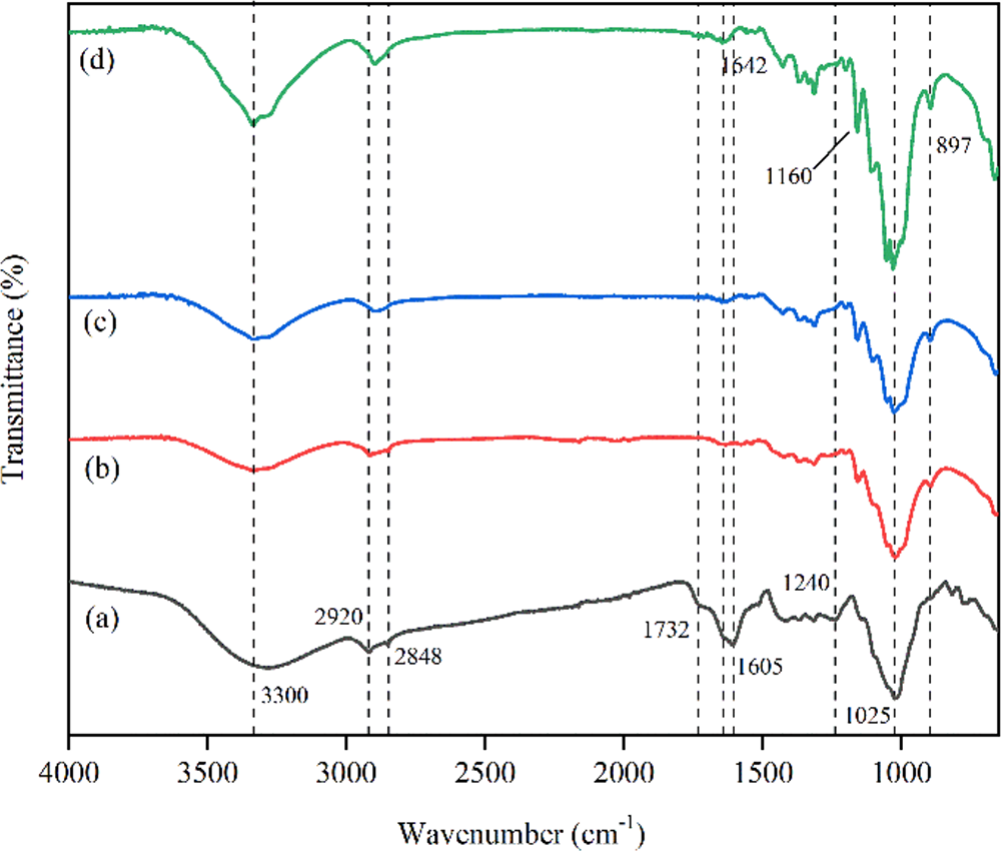
FT-IR spectra of (a) coffee pulp (CP), (b) alkali-treated CP (A-CP), (c) bleached CP (B-CP), and (d) nanocellulose CP (NCP).

FTIR spectroscopy was used to investigate the chemical structural changes of CP after various chemical treatments based on the characteristic frequencies of their molecular functional group vibrations.^25^ As shown in Figure 5, the spectral difference is attributed to the changes in the compound sample after various chemical treatments. In the region at 3400–3100 cm^–1^, all samples showed stretching vibrations of the OH group in cellulose molecules. The band at around 2920–2800 cm^–1^ presents a C–H stretching vibration. The spectra around 2848 cm^–1^ contributed to C–H stretching in aliphatic wax fractions.^26^ The peak intensity around 2920 cm^–1^ of B-CP and NCP declined due to the removal of lignin components during chemical treatment as cellulose. The band at 1732 cm^–1^ was present only in CP, which was assigned to the C=O stretching vibration of the acetyl and ester groups of pectin, hemicellulose, or the carboxylic acid groups ferulic and *p*-coumaric of lignin.^27^ The C=O stretching disappeared in A-CP, B-CP, and NCP since the carboxylic groups were eliminated after the alkali treatment. In addition, traces of fatty acid on the fiber surface were also removed.^28^ Also, the peak at 1240 cm^–1^disappeared in the sample after NaOH and NaClO_2_ treatment and sulfuric acid hydrolysis. This peak was attributed to the stretching vibration of the C–O group of the acetyl or aryl group in lignin.^29^ The region between 1641 and 1649 cm^–1^ was attributed to O–H bending of absorbed water that appears in all samples.^30^ Bands at 1605 cm^–1^ (C=C stretching vibration of the aromatic ring in lignin) were observed in CP. These bands were verified to be weakened after chemical treatment. The C–O–C pyranose ring stretching of cellulose was observed in the range of 1020–1032 cm^–1^.^31^ Meanwhile, the small peak around 897 cm^–1^ was associated with the C–H bending vibrations and C–O–C stretching vibrations of β-glycosidic linkages in cellulose. In the NCP sample, the peak at 1165 cm^–1^, represents the sulfate group which may be due to the sulfonation of cellulose occurring during sulfuric acid hydrolysis.^21^ The absence of characteristic peaks in B-CP and NCP proved that various chemical treatments could completely isolate B-CP and NCP from CP.

##### 3.2.2.2 X-Ray Diffraction Analysis

**Figure 6:**
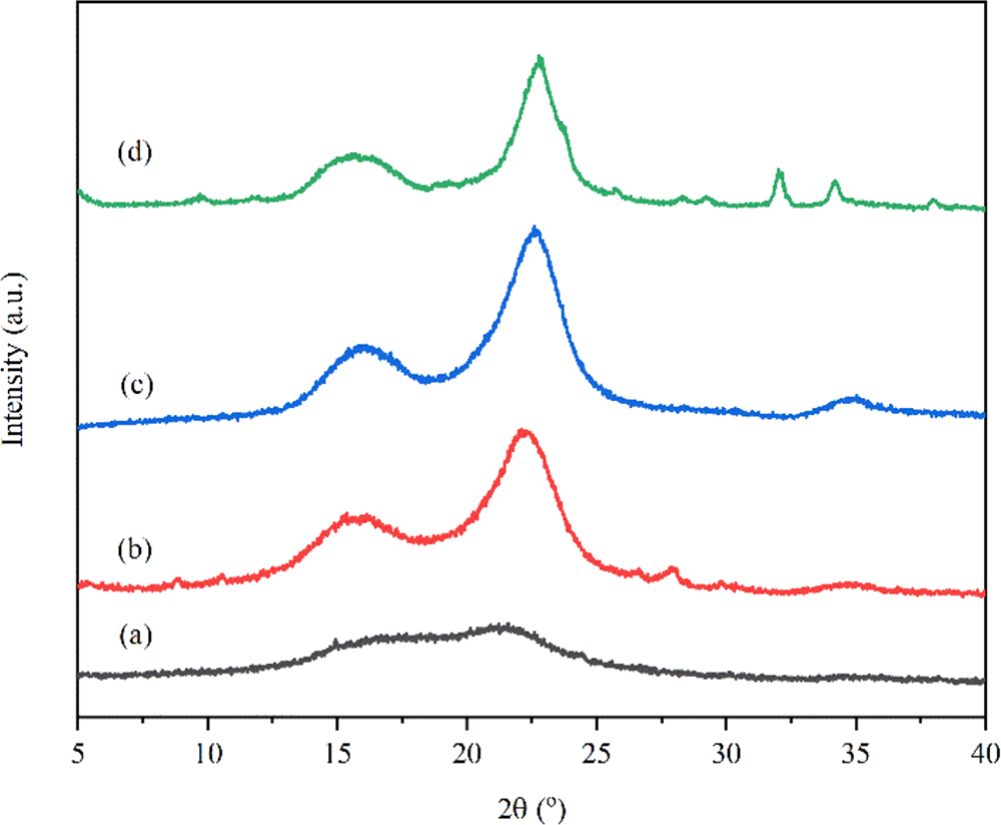
XRD patterns of (a) CP, (b) A-CP, (c) B-CP, and (d) NCP.

In general, cellulose consists of both crystalline and amorphous regions: lignin and hemicellulose. Figure 6 shows the X-ray diffraction patterns of CP and CP obtained after the chemical process. It can be observed that all samples of CP presented main intensity peaks at diffraction angles (2θ) of approximately 15°, 22°, and 34°, which correspond to the diffraction lattice planes (110), (200), and (400) of the typical cellulose I structure, respectively. Similar patterns of XRD indicated that the pretreatment of CP did not affect the natural structure of cellulose I. After hydrolysis, the intensity of NCP is lower than that of CP obtained from alkali and bleaching treatments^32^ as the crystallinity index (CI) of NCP was approximated to be 80.55% higher than that of B-CP (68.89%), A-CP (56.94%), and CP (20.17%). These results implied that the noncellulose components and amorphous regions in the CP fiber were eliminated during the chemical process. The crystal size value revealed in Table 2 was determined to be perpendicular to the plane (200). It was found that the crystal size was increased from 0.80 to 3.94 nm after chemical treatments, which is due to the coalescence of smaller crystalline domains.^32,33^

**Table 2:** Crystallinity Index of CP at Different Treatments

sample	2θ amorphous (°)		2θ (200) (°)		crystal size (nm)	crystallinity index (%)
	degree	intensity	degree	intensity		
CP	18.77	1880	21.57	2355	0.80	20.17
A-CP	18.40	2194	22.09	5095	2.09	56.94
B-CP	18.40	1782	22.60	5728	2.68	68.89
NCP	18.51	821	22.77	4222	3.94	80.55

#### 3.2.3 Thermal Properties of Prepared Nanocellulose

**Figure 7:**
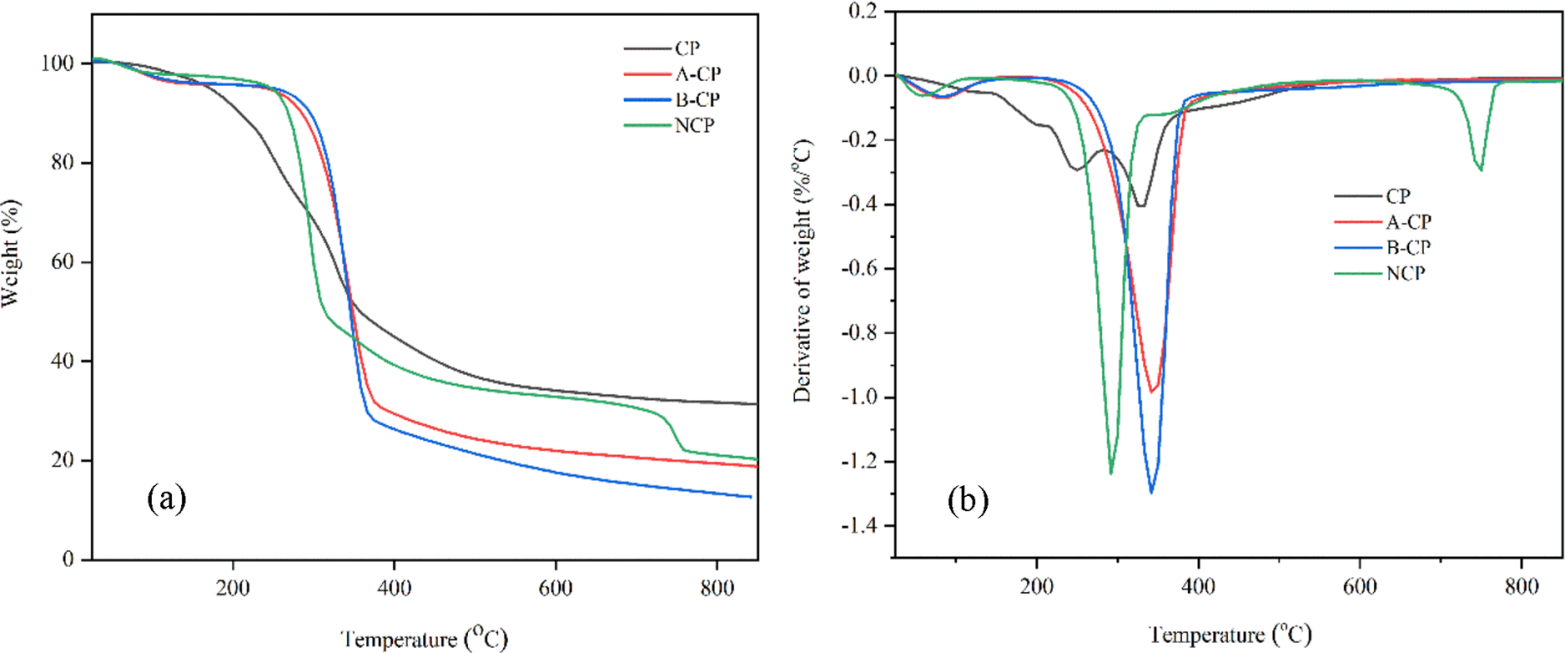
Thermal analysis of CP, A-CP, B-CP, and NCP: (a) TGA curve and (b) DTG curve.

Thermogravimetric (TG) and derivative thermogravimetric (DTG) curves of CP, as well as those obtained after alkali treatment, bleaching, and hydrolysis, are presented in Figure 7a,b. The samples’ thermal decomposition behavior showed two main mass loss stages: (i) low temperatures, below 150 °C and (ii) temperatures from 150 to 850 °C. Table 3 summarizes the thermal degradation of CP via different treatments from the extracted curve. In the range of 25–150 °C, all samples appeared to have a slight weight loss (less than 5%) due to the evaporation of water from the surface fibers or low-molecular-weight compounds.^34^ In the range of 150–850 °C, the weight loss would be greater (more than 60%), due to lignocellulose degradation, than that they have degraded in temperatures between 150 and 500 °C.^20^ The thermal decomposition of hemicellulose begins at 150 °C and continues to 350 °C. Cellulose decomposed mainly in the temperature range of 275–350 °C. Meanwhile, lignin decomposition occurs at a wide temperature range from 250 to 500 °C.

**Table 3:** Degradation Temperature of CP, A-CP, B-CP, and NCP

material	*T*_onset_ (°C)	*T*_max_ (°C)	weight loss (%)	residue (%) at 850 (°C)
CP	150.96	333.33	65.31	31.39
A-CP	208.33	341.67	77.39	18.83
B-CP	216.67	341.67	83.01	12.66
NCP	177.92	291.67	63.71	20.30

From the DTG curve, it is observed that several peaks appeared only in CP : at 125, 200, 250, and 333.33 °C, some of which were attributed to light volatile materials or noncellulose contents.^35^ Peaks at 200, 250, and 333.33 °C correspond to hemicellulose, lignin, and cellulose decomposition, respectively. The peaks at the 125–250 °C region disappeared during alkali and bleaching treatments, indicating that hemicellulose and a fraction of lignin were loosened from CP. After the bleaching treatment, temperature degradation increased significantly due to the elimination of the noncellulose composition and the higher cellulose content. The most weight loss occurred when b-CP initially degraded at 216.67 and 341.67 °C.

The initial degradation of NCP, produced after hydrolysis by 65 wt % sulfuric acid for 1 h, begins at 177.92 °C, with the maximum degradation peak at 291.67 °C observed to be lower than that of the treated CP. This was attributed to the surface sulfation from sulfuric acid treatment where the sulfate group (SO_3_^–^) was used as a substitute for the hydroxyl groups (−OH) of the cellulose structure during acid hydrolysis.^20,21^ Therefore, the activation energy of degradation of NCP was reduced, and NCP is also less resistant to pyrolysis. As a result, the hydrolysis treatment led to an increase in char fraction compared to alkali and bleaching treatments. Additionally, the lower stability may be attributed to the smaller dimensions of NCP, which give a higher surface area of NCP to expedite the heat transfer process and degradation rate.^25^

### 3.3 Fabrication of Nanocellulose-Based PVA Composite Film

To fabricate a composite film, NCP was used as a nanofiller in the PVA matrix to obtain PVA/NCP nanocomposites fabricated via the solution casting method. The effect of different contents of NCP (1, 3, and 5 wt % NCP) with and without glycerol (25 wt % related to mass PVA) on their properties, including chemical, optical, physical, and thermal properties, was observed.

#### 3.3.1 Chemical Structural Changes of Composite Films

**Figure 8:**
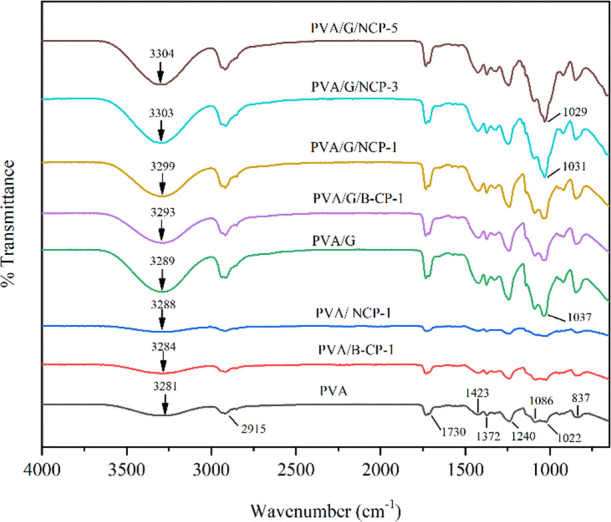
FT-IR spectra of neat PVA, PVA/B-CP, and PVA/NCP with glycerol and without glycerol nanocomposites.

The interfacial compatibility and miscibility of the matrix and filler components can define the structure and properties of nanocomposite materials. The chemical structural change of nanocomposite films between PVA and B-CP or NCP (with and without glycerol) was determined by FT-IR spectra, as shown in Figure 8. In neat PVA, the hydroxyl, methyl, and acetate groups were the main characteristic peaks, obviously in visible spectra. O–H stretching and hydroxyl group (−OH) bending vibration peaks occurred at 3281 and 1423 cm^–1^. The peak at 2917 cm^–1^ was assigned to the asymmetric stretching vibration of the methyl group (CH_2_). A vibration peak at about 1730 cm^–1^ corresponded to the stretching vibrations of C=C and −C–O from the residual acetate groups in the PVA matrix.^36,37^ C–O stretching of acetyl groups in the PVA backbone was also detected at a peak around 1086 cm^–1^, and O=C–O–C stretching of acetate occurred at the 1240 cm^–1^ band. The peak at 1372 cm^–1^ was assigned to the bending vibration of C–H bonds. Meanwhile, band 837 cm^–1^ was assigned to the rocking of −CH_2_.^38^ A peak at about 1645 cm^–1^ represented almost all spectra assigned to the H–O–H bending of adsorbed or bound water.^39^ For neat PVA, a slight peak can be observed at 1646 cm^–1^. It is well known that the hydroxyl band is sensitive to hydrogen bonding and can be compelled to shift the wavenumber in FT-IR spectra; adding glycerol and B-CP or NCP into the PVA matrix led to a slight shifting of hydroxyl bands. The addition of plasticized glycerol into the PVA matrix showed that the band related to the vibration stretching of −OH was broad and large. Moreover, this band shifted from a higher wavenumber of 3281–3289 cm^–1^ and became sharper with glycerol, proving that glycerol could switch the hydrogen bonding in PVA or PVA/B-CP or NCP composite.^40^ After adding glycerol, the intensity of all peaks appeared to increase or become stronger due to the hydrophilic compound. In addition, it indicated a good mix in the blend.^41^

Comparing the nanocomposite PVA between B-CP and NCP, it was found that their infrared spectra were similar. The addition of B-CP and NCP in the PVA matrix with glycerol and without glycerol resulted in the intensity of the −OH stretching vibration, owing to the strong intermolecular interaction in this composite, as a result of interaction between the −OH group on the surface of cellulose or nanocellulose and the macromolecular chain of PVA. It could be confirmed by the slight shifting of the centered wavenumber compared to neat PVA. At a centered peak in the range of 2916–2918 cm^–1^ were assigned C–H stretching vibrations from the alkyl group, as seen in all samples. Bands at 1239–1243 cm^–1^ were attributed to the C–O stretching vibrations. These peaks’ intensities were likely to reduce after the addition of B-CP or NCP due to the formation of hydrogen bonds in PVA and B-CP or NCP.^42,43^ The peak in the range of 837–846 cm^–1^ in all samples was likely to decrease with the addition of NCP or B-CP, and this phenomenon probably supported the interaction of PVA and nanocellulose.^37^ However, it could be observed that the peak at around 1030 cm^–1^ with the addition of 3 and 5 wt % NCP had higher intensity than PVA/G, as this band corresponded to the C–O–C pyran ring stretching vibration of aliphatic primary and secondary alcohols in nanocellulose.^44^

#### 3.3.2 Optimal Properties of Composite Films

**Figure 9:**
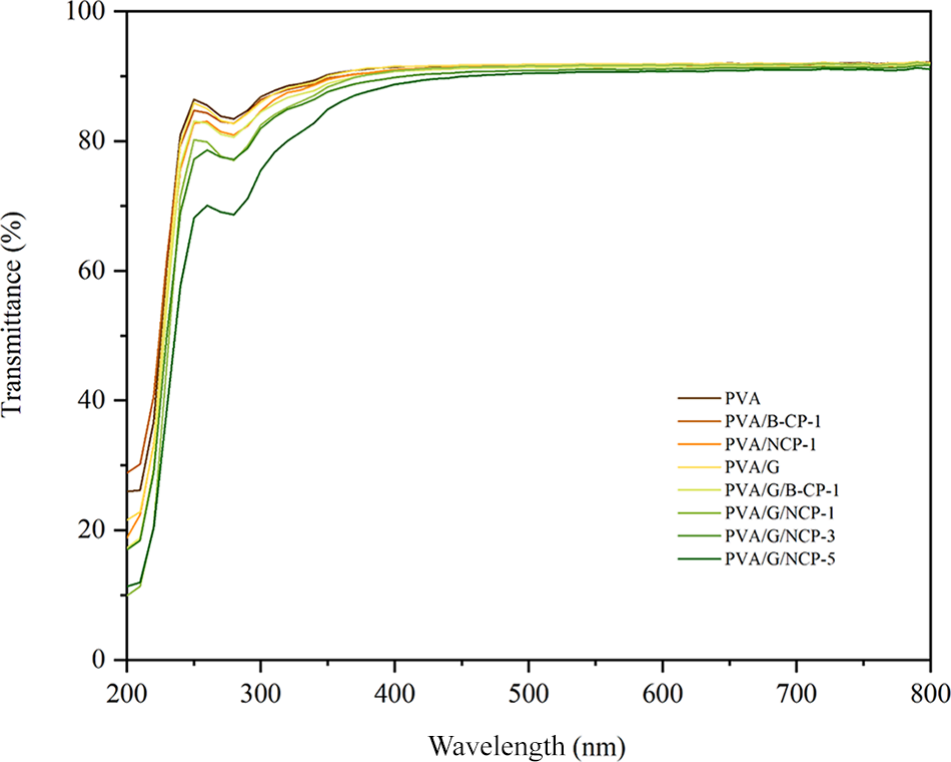
UV–vis transmittance of PVA composite films.

Transparency is a desirable film characteristic for packaging. It is a criterion for the dispersion of cellulose or nanocellulose in a polymer matrix, in which the transparency level of composite films was investigated using UV spectroscopy. Figure 9 shows the percent of transmittance in the UV–vis range of 200–800 nm of neat PVA and PVA/B-CP or NCP composite films with and without a glycerol plasticizer. It is well known that PVA shows good transmittance in the visible wavelength range (92.20% at λ = 800 nm) and has good film-forming properties that can be utilized in various applications.^43^ The addition of 25 wt % of glycerol in the PVA matrix shows that transparency at λ = 800 nm did not change significantly. However, it slightly decreased from 92.20 to 92.05. The reduction in the PVA transmittance is probably because of scattering, resulting from the random distribution of the crystalline domain on PVA/glycerol films, and indicated that glycerol could be miscible in the PVA matrix.^45^ At a wavelength of 800 nm, the transmittance percent of PVA/B-CP and PVA/G/B-CP was 91.72 and 91.75%, respectively, and the transmittance percent of PVA/NCP and PVA/G/NCP composites with the NCP content of 1, 3, and 5% was 91.97, 91.85, 91.70, and 91.06%, respectively. This result indicated that incorporating B-CP or NCP in the PVA matrix did not largely impact the film transparency. All PVA/B-CP and PVA-NCP composites with glycerol and without glycerol presented the same transparency level with a very slight decrease of transmittance percent at λ = 800 nm, compared with neat PVA. It confirmed that B-CP or NCP was well dispersed in the PVA matrix and compatible with blending PVA.^46^ This transmittance result may confirm the fabrication efficiency of PVA/B-Cp or NCP composite films to be used as active materials in packaging.

#### 3.3.3 XRD Analysis of Composite Films

**Figure 10:**
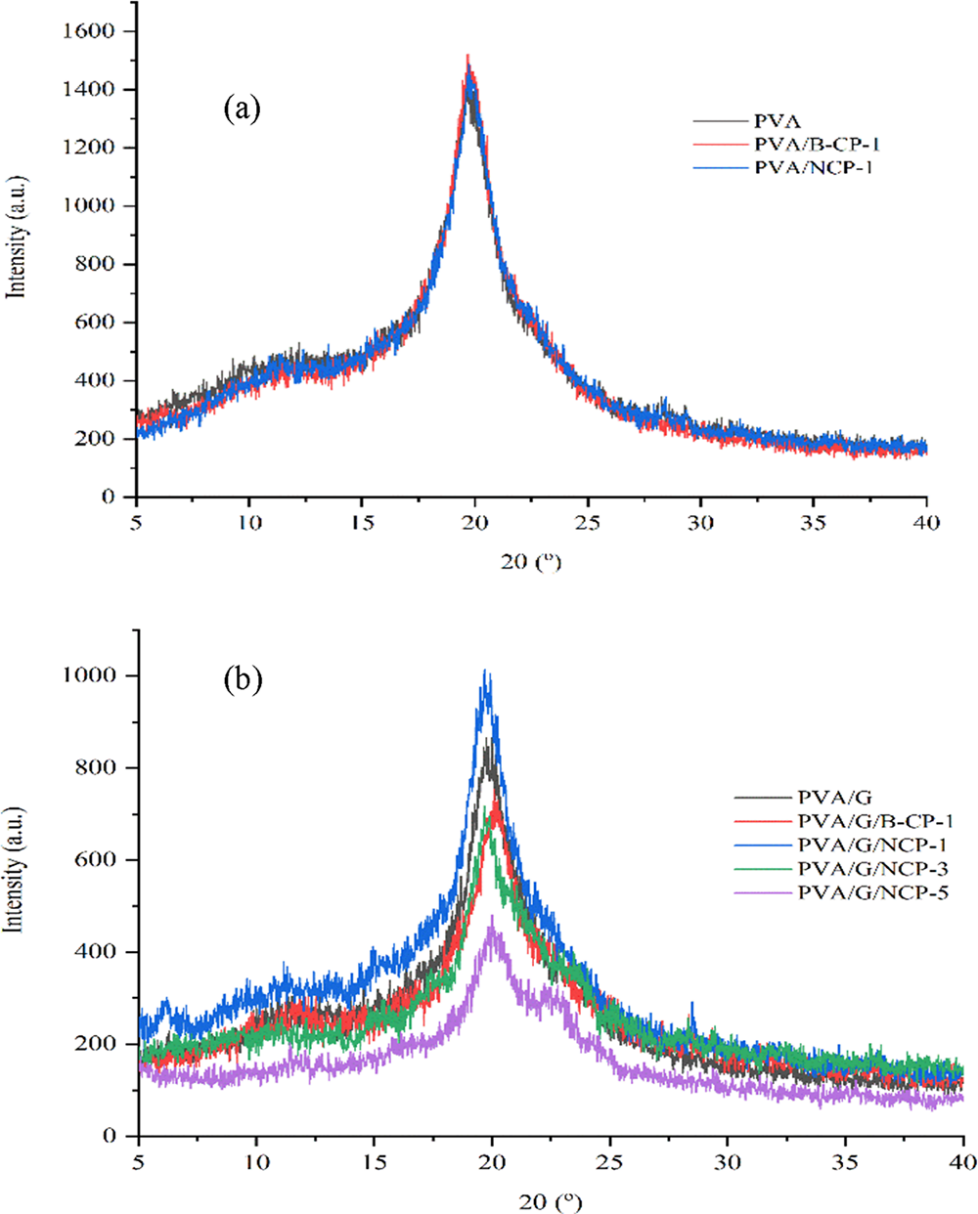
XRD patterns of the PVA composite: (a) without glycerol and (b) with glycerol.

The crystalline structure of neat PVA, PVA/B-CP, or NCP composite films with and without glycerol was studied using XRD in the 2θ range of 5–40°. The XRD patterns of PVA and PVA/B-CP or NCP composite are shown in Figure 10. The diffraction peak of neat PVA exhibited a typical crystalline peak at 19.66° corresponding to the (101) plane of semicrystalline PVA. This characteristic peak also appeared in the diffraction pattern of PVA/B-CP or NCP composite with glycerol and without glycerol, indicating that the crystal structure of PVA was maintained even with the addition of cellulose or nanocellulose.^47,48^Figure 10a displays the diffractograms of neat PVA, PVA/B-CP, and PVA/NCP (1 wt % of B-CP or NCP) composites, from which it could be observed that these characteristic peaks had superposition. Incorporating B-CP or NC into the PVA matrix slightly increased the intensity of the (101) plane. Moreover, the absence of characteristic peaks of B-CP and NCP corresponds to the homogeneous dispersion into the PVA matrix and strong interaction among PVA and B-CP or NCP.^49^

Figure 10b shows the effect of glycerol as a plasticizer on the crystalline structure of PVA. It was found that the diffraction peak at around 19.77° corresponding to neat PVA weakened with the addition of 25 wt % of glycerol, suggesting a reduction of the crystalline phase of PVA/G due to a decrease of intra- and intermolecular interactions of the PVA chain.^50,51^ From Figure 10b, it is observed that the diffraction of PVA/G/B-CP and PVA/G/NCP (with 1–5 wt % content of NCP) composites showed superposition of the characteristic peaks of the two components. The peaks at approximately 2θ = 22.60° and 22.77° of B-CP and NCP, respectively, were attributed to the (200) plane of cellulose I, as shown in Figure 6. The intensity of the diffraction peak of NCP became more pronounced with the addition of more than 1 wt % of NCP. Then, this peak was likely to decrease with the addition of NCP.

Meanwhile, the intensity of diffraction peaks of the PVA/G/NCP composite at about 19.77° became lower and narrow with an increased NCP content. There is a possibility that the NCP can interrupt the regular packing of the molecule chain of PVA, and the presence of NCP or B-CP causes restricted mobility of the PVA chain by hydrogen-bond formation at the interface.^52,53^ The effect of glycerol and B-CP or NCP content also influences the mechanical and thermal properties.

#### 3.3.4 Thermal Behavior of Composite Films

**Figure 11:**
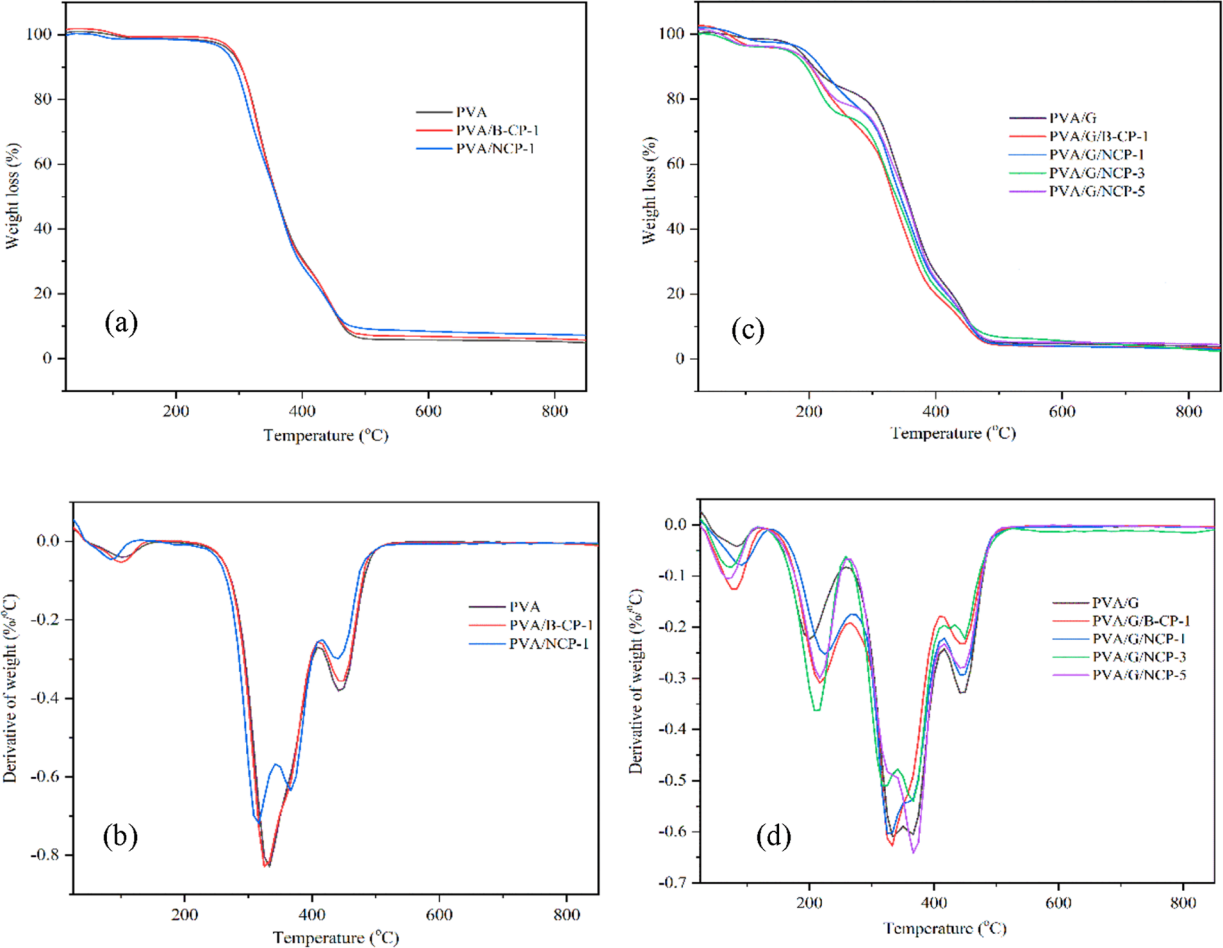
TGA and DTG curves of the PVA composite (a, b) without glycerol and (c, d) with glycerol.

TGA was used to evaluate the thermal behavior of PVA-based composites to know the thermal stability and degradation of PVA-based composites, as affected by the addition of B-CP, different NCP contents, and glycerol as a plasticizer (Figure 11). The presence of three main degradation steps of PVA itself and PVA-based composites was explicit from the derivative thermogravimetric (DTG) curve of weight loss profiles, as shown in Figure 11(b,d). All samples presented a similar initial weight loss in the temperature region of 30–140 °C due to the evaporation loss of physically weak and loosely bound moisture on the surface of composite films. The weight loss in these regions was less than 5 wt %.^37^ The second region of weight loss occurred over 200–400 °C, as the weight loss in range was about 70 wt %. This loss was structural degradation of PVA via dehydration accompanied by the formation of some volatile products. The third region of weight loss occurred above 400 °C, involving the degradation of PVA for main-chain decomposition and decomposition of the carbonaceous matter.^16,54^ From Figure 11a,b, the incorporation of 1 wt % of B-CP or 1 wt % of NPC could be observed in the PVA matrix, The maximum degradation rate of temperature (*T*_max_) of PVA composites decreases, as a result of the thermal behavior of B-CP and NCP, both of which were reported previously. The *T*_max_ value was 329.27, 326, and 313.33–368.83 °C for PVA, PVA/B-CP-1, and PVA/NCP-1, respectively. It was reported that the increasing char residue resulting from CNC pyrolysis was catalyzed by the acid sulfate group.^55^ Herein, the amount of char residue of PVA/NCP-1 (7.21 wt %) provided a higher content than neat PVA (4.91 wt %).

Incorporating glycerol at 25 wt % in the PVA matrix induced a new peak at about 198 °C, as shown in Figure 11c. The decomposition temperature occurred at 190–250 °C, resulting from the decomposition of glycerol.^56^ When comparing PVA/G with neat PVA, it was observed that the onset decomposition temperature (*T*_onset_) and the maximum degradation rate of temperature (*T*_max_) of PVA/G were higher than that of pure PVA, resulting in high thermal stability and indicating the good interaction between glycerol and the PVA matrix, corresponding to FT-IR and UV–vis characterizations. Moreover, the temperatures of B-CP and NCP based on PVA composites with the incorporation of glycerol also shifted to higher *T*_onset_ and *T*_max_ than those without glycerol. However, the addition of more than 1 wt % of NCP resulted in a slight decrease in thermal stability since a higher fiber content was probably aggregated, leading to a lower interfacial area between the PVA matrix and NCP as the filler.^57,58^

#### 3.3.5 Mechanical Properties of Composite Films

**Figure 12:**
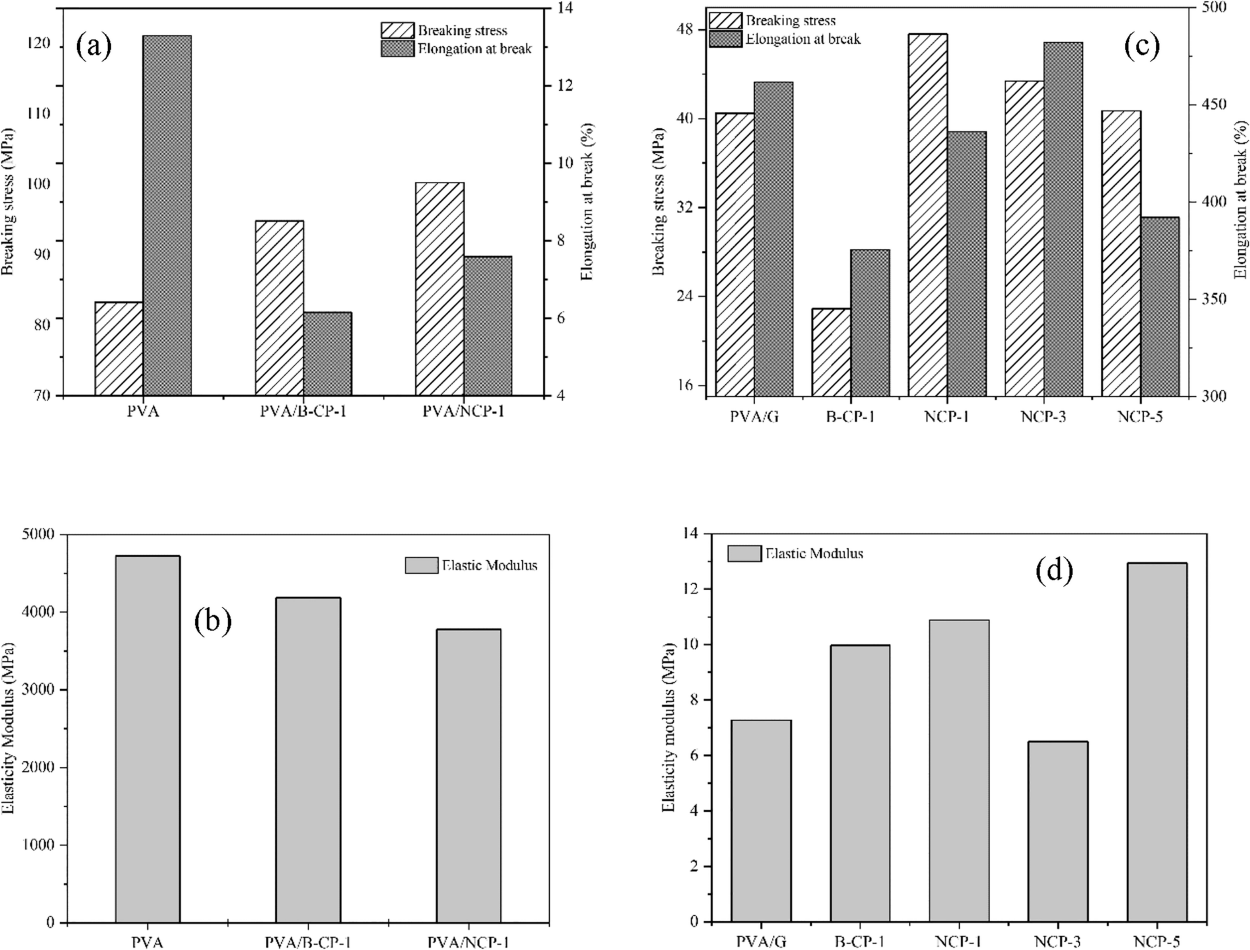
Breaking stress, elongation at break, and elasticity modulus of PVA composites: (a, b) without glycerol and (c, d) with glycerol.

The mechanical behavior of PVA composite films was significant to characterize their strength and durability to resist extraneous factors. Herein, mechanical properties, including breaking stress, elongation at break, and elasticity modulus, were characterized by a universal tensile machine (UTM), as shown in Figure 12. For neat PVA, the breaking stress (BS), elongation at break (EB), and elasticity modulus (EM) were 83.30 MPa, 13.29%, and 4721.25 MPa, respectively. From Figure 10, it is clearly observed that the addition of B-CP and NCP into the PVA matrix improved the breaking stress or tensile strength. The 1% NCP-loaded PVA provided tensile strength higher than 1% B-CP-loaded PVA, which was 100.25 and 94.80 MPa, respectively, due to the high surface area of the NCP.^59^ Furthermore, the formation of the percolation network of NCP from interaction among the NCP by intra and intermolecular hydrogen bonding between NCP and PVA matrix may be another reason for higher tensile strength.^15^ However, the elongation at break of PVA composites decreased with the addition of B-CP or NCP, 6.15% for PVA/B-CP-1 and 7.59% for PVA/NCP-1.

By adding glycerol as a plasticizer into the PVA matrix to improve their brittleness, it was observed that glycerol plays a role in the mechanical properties of the PVA composite. The elongation at break increased to 461.67%, and tensile strength and elastic modulus decreased to 40.5 and 7.27 MPa, respectively; the PVA structure became more flexible with the addition of glycerol since glycerol reduced the inter–intramolecular force of the PVA chain, leading to a lower tensile strength and elastic modulus. In contrast, the elongation at break was higher.^60^

The effect of NCP content on the breaking stress, elongation at break, and elastic modulus of the PVA nanocomposite with glycerol is shown in Figure 12c,d. The tensile strength of the PVA nanocomposite increased with the filler NCP content. The highest tensile strength value was obtained at 1 wt % of NCP, which was 47.61 MPa, indicating good reinforcement in the PVA nanocomposite. However, the higher tensile strength with 1 wt % NCP slightly declined to 43.39 (3%) and 40.69 (5%) MPa. It was ascribed to the aggregation of NCP in the PVA matrix at a high-level content,^59^ and the corresponding intensity of the crystalline structure of the PVA composite became lower, as shown in Figure 10c. Moreover, the elastic modulus increased with the addition of NCP content from 7.27 to 12.94 MPa. In contrast, addition of NCP reduced the elongation at break, from 461.67% for PVA/G to 392.1% for the PVA/G/NCP-5 nanocomposite. The decrease in elongation at break may be due to the restricted mobility of the PVA chain due to the enhancement of the film’s stiffness.^61,62^ Therefore, the result of the PVA nanocomposite mechanical behavior was improved at lower NCP contents.

## 4 Conclusions

In this study, the CP was subjected to alkali and bleaching treatments, followed by acid hydrolysis for nanocellulose (NCP) preparation. The result revealed that the noncellulose, including lignin and hemicellulose, was successfully eliminated after pretreatment the alkali and bleaching treatment. The physicochemical characterization results of NCP showed increases in the crystallinity index from 20.17% (CP) to 80.55% (NCP), indicating the removal of the amorphous phase of cellulose. The obtained NCP has a rodlike or needle-like shape with an average diameter of 16.03 ± 4.70 nm. The obtained NCP was used as a nanoreinforcement of the PVA film via the solvent casting method to improve the PVA properties. Incorporating different NCP contents (1, 3, and 5 wt %) into PVA exhibited that the NCP filler had significantly improved properties of nanocomposite PVA films. The physicochemical characterization results showed better crystallinity and tensile strength than that of PVA/G films. However, their properties declined beyond 1 wt % of NCP content due to the aggregation of NCP in the PVA matrix. On the other hand, incorporation of various NCP contents showed a slightly significant percentage of transmittance.
